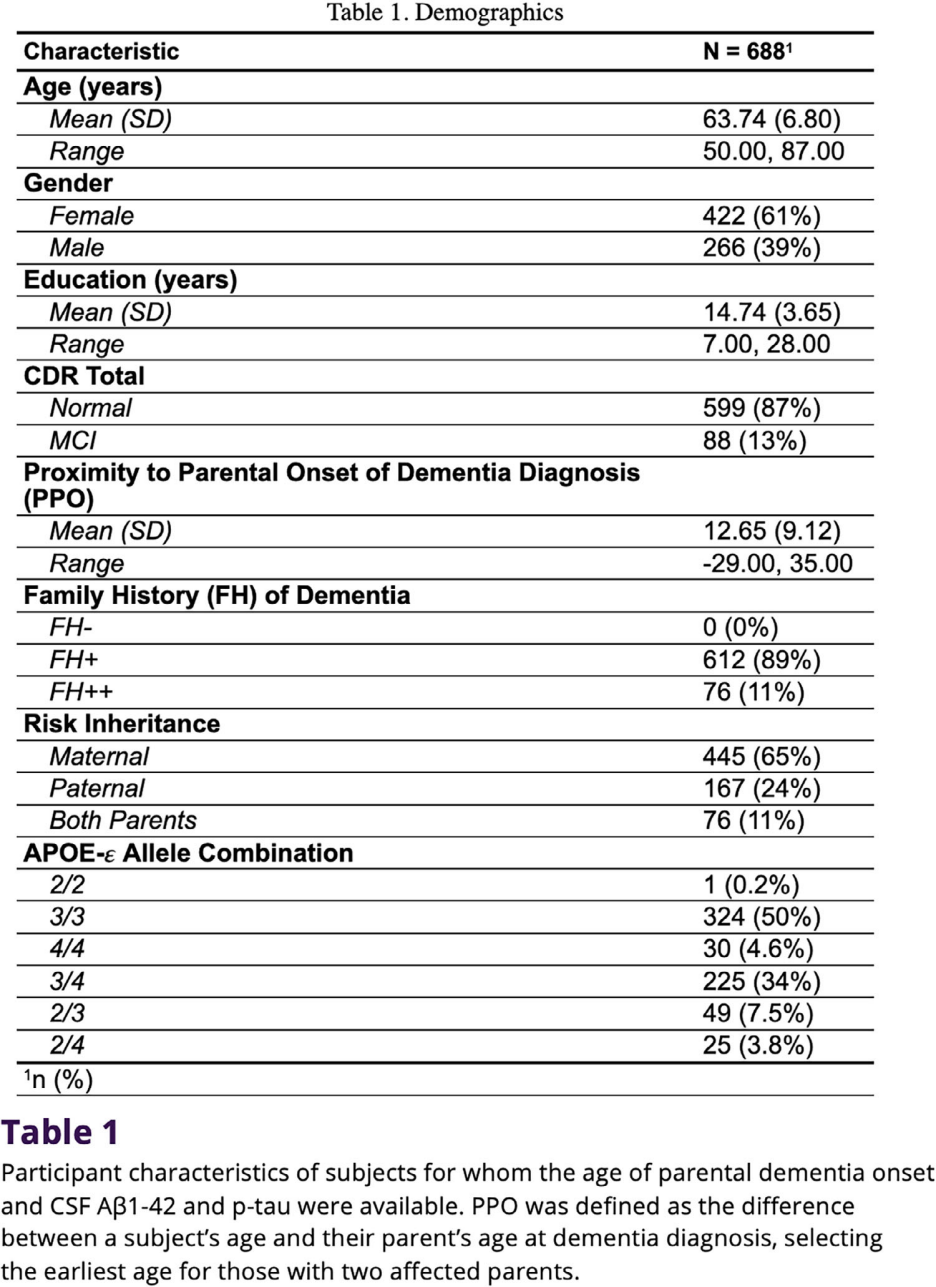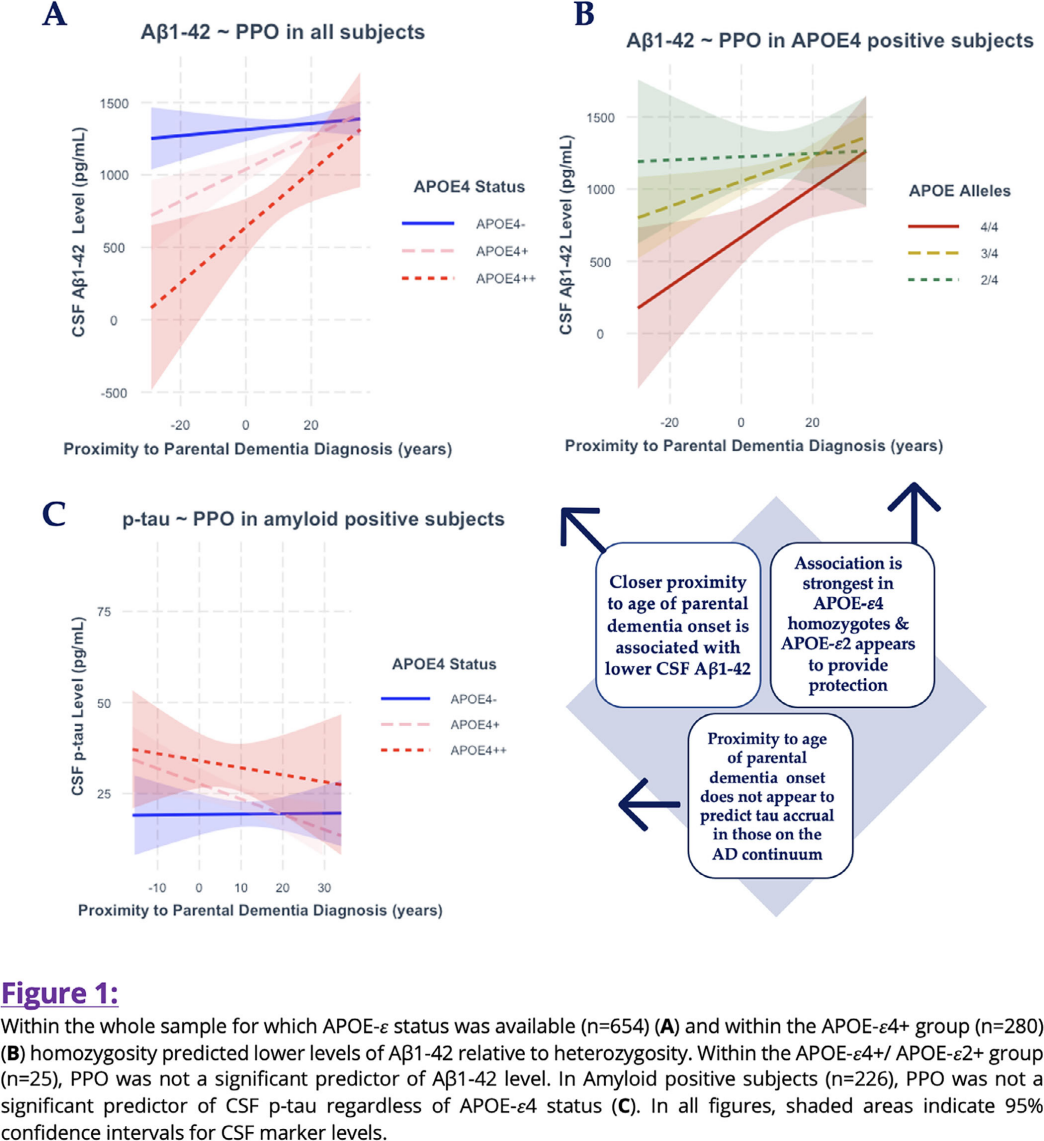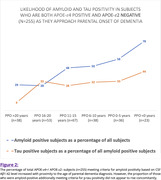# Accrual of Alzheimer’s Disease pathology as a function of proximity to parental dementia onset

**DOI:** 10.1002/alz.091192

**Published:** 2025-01-09

**Authors:** Elina T Ziukelis, Elijah Mak, Craig W Ritchie, John T O'Brien, Dag Aarsland

**Affiliations:** ^1^ King's College London, London, London UK; ^2^ South London and Maudsley NHS Foundation Trust, London UK; ^3^ Department of Psychiatry, University of Cambridge, Cambridge UK; ^4^ Scottish Brain Sciences, Edinburgh UK; ^5^ Edinburgh Dementia Prevention, University of Edinburgh, Edinburgh UK; ^6^ University of Edinburgh, Edinburgh UK; ^7^ UK Dementia Research Institute, Institute of Psychiatry, Psychology & Neuroscience, King's College London., London UK

## Abstract

**Background:**

There is emerging evidence to substantiate temporal proximity to parental onset (PPO) of dementia as a proxy stage marker in studies of late onset Alzheimer’s Disease (AD). PPO predicts accrual of amyloid pathology cross‐sectionally and longitudinally. However interactions with gender, age, and APOE‐𝜀4 carriage have been inconsistent across datasets and amyloid measures.

**Method:**

We investigated cross‐sectionally a subset of the European Prevention of Alzheimer's Dementia (EPAD) study cohort (n=688) (Table 1). PPO was calculated by substracting a subject’s age from the youngest age of diagnosis of an affected parent. Regressions were used to determine whether PPO predicts CSF fluid Aβ1‐42 in the whole sample or p‐tau in an amyloid‐positive subgroup (n=226). Possible interactions with age, gender, education, APOE‐𝜀 status, family history load, gender of affected parent and CDR score were explored.

**Result:**

Nearer PPO predicted lower CSF Aβ1‐42 level (p<0.001), interacting significantly with APOE‐𝜀4 carriage only (β=12.683; T=3.295; p=0.001) accounting for effects of age, gender and education level. In APOE‐𝜀4+ subjects, APOE‐𝜀2 provided a protective effect (Figure 1). At least half of APOE‐𝜀4+/APOE‐𝜀2‐ subjects (n=255) within ten years of age of parental onset were amyloid positive (Figure 2). PPO did not predict p‐tau levels in amyloid positive subjects (n=226) (p=0.414).

**Conclusion:**

In APOE‐𝜀4+/APOE‐𝜀2‐ individuals, PPO predicts accrual of amyloid but not tau pathology, independently of age. Selectively examining this population as they approach age of parental onset may better elucidate the natural history of preclinical late onset AD.